# What should a Universal School-Based Psychoeducational Programme to Support Psychological Well-Being amongst Children and Young People in South Africa Focus on and how should it be Delivered? A Multi-Stakeholder Perspective

**DOI:** 10.1007/s12310-021-09465-3

**Published:** 2021-07-13

**Authors:** Bronwynè J. Coetzee, Hermine Gericke, Suzanne Human, Paul Stallard, Maria Loades

**Affiliations:** 1 Department of Psychology, Stellenbosch University, Stellenbosch, South Africa; 2 Department for Health, University of Bath, Bath, UK; 3 Department of Psychology, University of Bath, Bath, UK

**Keywords:** Children and young people, Depression, Anxiety, School-based, Prevention, South Africa

## Abstract

Children and young people are vulnerable to developing mental health problems. In South Africa, this vulnerability is compounded by contextual risk factors such as community violence and poverty. However, mental health services are scarce and costly, which precludes access for many. Universal school-based mental health programmes can prevent the onset of mental health problems in children and young people and have been implemented to good effect in high-income settings. We sought to understand stakeholder perspectives on what such a programme should focus on and how it could be implemented in practice within the South African context. We interviewed children and young people (*n* = 22), parents (*n* = 21), teachers (*n* = 17), and school mental health counsellors (*n* = 6) recruited from two schools in the Western Cape, South Africa. Interviews were audio-recorded, transcribed verbatim and analysed thematically. We generated three overarching themes: ‘the value of a mental health and well-being programme’, ‘content and delivery’, and ‘practicalities and logistics’. Participants were optimistic about the potential value of such a programme. Developing content that was appropriate for group delivery, flexible and timed to fit within the school schedule was important. Finding ways to make activities meaningful for large classes was important logistically, as was determining to what extent leaners would feel comfortable participating alongside their peers. Participants felt that outsiders, as opposed to school staff, should deliver the programme and that parents should be involved where possible. Developing a mental health programme for children and young people in the South African context requires careful understanding of who the key role players in such an intervention will be and how exactly they want to be involved and, how the challenges associated with practicalities and logistics can be overcome.

## Background

Globally, the rates of depression and anxiety amongst children and young people (CYP) are high and without intervention, often persist into adulthood (Costello et al., 2006; Woodward & Fergusson, 2001). The prevalence of mental health problems in CYP ranges between 10 and 20%, and at least 20% of CYP will have experienced a mental health issue by age 18 (Costello et al., 2003; Essau et al., 2012). Further, depression and anxiety are highly comorbid and estimates suggest that comorbidity ranges from 10 to 50% (Garber & Weersing, 2010).

In low- and middle-income countries (LMICs) CYP are at an even higher risk for developing these disorders due to the many risk factors associated with living in poverty and conditions of precarity (Kieling et al., 2011; Lund et al., 2011; Patel et al., 2010; Skeen et al., 2019). In South Africa, for example, available data is limited but suggests that rates are higher. For example, a 2001 study suggests that the prevalence of anxiety among children 7–13 years ranges between 22% and 25.6% (Perold, 2001). South Africa is characterised by high rates of violence, trauma and poverty and high rates of infectious diseases like HIV/Aids and tuberculosis (TB). Each of these risk factors make children living in this country vulnerable to a spectrum of psychological problems. Given the onset of mental health disorders during childhood, the heightened risk factors, and the relative absence of sufficient service provision to address these issues in South Africa (Kieling et al., 2011; Lund et al., 2011; Patel et al., 2010; Skeen et al., 2019), there is an urgent need to focus on the prevention of mental health disorders, as there is evidence of both short-term and long-term consequences of untreated mental health problems.

In the short term, mental health problems in CYP have been shown to impact on daily functioning, disrupt educational attendance and attainment, affect social relationships and interfere with normative development (Belfer, 2008; Finning et al., 2019; Giel et al., 1981; Patel et al., 2007; Rescorla et al., 2007; Wickersham et al., 2019). In the long term, untreated depression is associated with an increased risk of subsequent depression, multiple health-risk behaviours (e.g. substance abuse), impacts on physical health, interpersonal difficulties, and self-harm and suicide (Ames & Leadbeater, 2018; Patel et al., 2007; Yu et al., 2017). These short- and long-term consequences of mental health problems make it even more important to prevent mental health disorders and build resilience wherever possible.

School-based prevention interventions which are ‘universal’ (that is, interventions which are delivered to all CYP, irrespective of whether they are at particular risk of developing mental health problems or are presenting with or experiencing symptoms of anxiety or depression or not) are showing promise (Waldron et al., 2018). Much of the available evidence about the effectiveness of school-based universal prevention programmes is derived from high-income settings like Australia, the USA and Canada. There have been mixed findings regarding effectiveness, and also few programmes have demonstrated sustained effectiveness at long-term follow-up (Mackenzie & Williams, 2018). A recent review of the effectiveness of 12 universally delivered programmes in the United Kingdom (Mackenzie & Williams, 2018) found that the interventions were delivered by a range of facilitators including school staff, psychologists and external facilitators often to different effects. For example, one study reported better outcomes for a cognitive behavioural therapy-based (CBT-based) prevention programme when it was delivered by health professionals as compared to teachers (Stallard et al., 2014). As such, the question of ‘who’ should deliver the programme remains an important one. While understanding ‘who’ should be delivering such programmes is important, it is equally important to determine how exactly these role players wish to be involved in the delivery of these programmes and this includes the extent to which parents should and want to be included in such programmes, which is likely to be context dependent.

However, evidence of the effectiveness of school-based programmes in LMICs is lacking. Our recent systematic review of school-based universal prevention programmes in LMICs identified 12 studies, which offered limited evidence for effectiveness (Bradshaw et al., 2021). This existing evidence suggested that children may benefit from such interventions especially when delivered in group format. Further, we concluded that the effectiveness of these programmes and the evidence for them may be improved through better and more careful implementation and comprehensive reporting. Long-term sustainability, cost-effectiveness, buy-in and involvement from stakeholders are key to positive outcomes (Kieling et al., 2011). As such, the design and adaptation of effective preventive programmes requires community ownership, cultural flexibility and fit with the delivery context to maximise effectiveness, appropriate training and support, and relevance and acceptability to stakeholders (Kieling et al., 2011). This is particularly important in a multicultural and multilingual country like South Africa.

As part of a larger, two-phased study on developing and assessing a prevention programme for young adolescents in a South African setting (Trial ID: PACTR202004803366609), we sought to explore the views of relevant stakeholders about how a universal school-based mental health programme within a particular South African setting should be delivered and what it should focus on. We aimed to explore the perspectives of CYP (10–15 years), parents/caregivers, teachers and school mental health counsellors.

## Method

### Research Setting

This qualitative study took place at two government funded primary schools in the Western Cape region of South Africa, in collaboration with a non-government organisation (NGO), who provides psychological support to learners in both schools. Both schools are urban, public schools and have a staff to learner ratio of more or less 40:1. Both schools form part of South Africa’s National School Nutrition Programme, which was introduced in 1994 (Devereux et al., 2018). Schools are eligible for funding for this programme when most of the learners come from low socio-economic status families. In total, each school has approximately 30–34 teachers and 900–1000 pupils. As part of the larger study, the two schools were randomly chosen (using computer randomisation) from a list of schools (*n* = 21) within which the NGO operates.

### Participants and Procedure

#### Participants

We used convenience sampling to recruit participants from the following key stakeholder groups:

1. CYP (male and female) in grades 5–7 (approximately 10–15 years of age) attending the selected schools;
2. Parents (also refers to, primary caregivers, carers or legal guardians who were 18 years and older) of CYP in grades 5–7 attending the selected schools;
3. Teachers of grades 5–7 learners at the selected schools; and
4. School counsellors working at the selected schools.

#### Recruitment Procedure

We invited learners in grades 5–7 as learners in these grades are between 10 and 15 years old, and are therefore cognitively and developmentally more mature than younger learners in the school and potentially better able to engage with abstract psychological concepts such as thoughts and feelings. We distributed an invitation letter explaining the purpose of the study to CYP at both schools. In addition, we also handed CYP an invitation letter to invite their parents to participate in the study. Across both schools, approximately 640 CYP were invited to participate. CYP interested in taking part were asked to obtain parental consent and return completed forms to either their teacher or a school counsellor. Interested parents were able to complete a contact permission form and return via their child to either a teacher or school counsellor.

Teachers and school counsellors at both schools were informed about the study by the project manager. Potential participants were able to contact the project manager directly to seek further information and to set up an interview. Participants for each of the groups were recruited consecutively until we had sufficient information to answer our research questions. That is, until information as it emerged from each of the groups became repetitive and suggestive of no new information (Francis et al., 2010).

#### Interview Development

Upon obtaining written informed assent and parental consent (CYP) or consent (parents, teachers and school counsellors), a short demographic questionnaire was completed, followed by a semi-structured interview. We developed an interview guide for each group of participants. As such, we developed an interview guide for CYP who participated, parents/caregivers, teachers and school counsellors. The interview guide for CYP was initially pilot tested with three learners and contained six different stories to elicit children’s responses on thoughts, feelings and behaviours. It has been shown that stories can be used as a child-friendly and non-threatening way of talking to children about abstract concepts (Human, 2018; Loxton & Human, 2017; Quakely et al., 2003; Quakley, 2001; Quakley et al., 2004). The purpose of the pilot test was to check whether children understood the interview questions and to limit ambiguity, to determine appropriate length of time of the interview, and to determine whether any additional questions needed to be added. Following the pilot interviews, we found that six stories were too many and decided on two stories to include in the final interview guide. In addition to these stories, we asked CYP what the words “feelings” and “thoughts” meant to them; what makes them feel happy, sad, angry, and scared; what they think when they experience these feelings; and what they do when they experience these feelings. We also asked CYP where and with whom they would feel most comfortable to talk to about their feelings; whether they would feel comfortable to talk about their feelings, thoughts and behaviours in front of their class; and who they think should do this type of a programme with them as a class.

We did not need to change the structure of our interview schedule with parents, teachers and school counsellors after initial pilot testing. We asked parents/caregivers in which ways they were involved in the school; what things we should focus on in the programme; whether they think that it is a good idea to implement the programme with entire classrooms; and if they would be interested in being part of it. We also asked parents/caregivers what various mental health terminology meant to them, and how they as a family unit manage emotions and mental health problems. We report on these responses in a separate paper.

We asked teachers whether they think that mental health problems is an issue amongst learners in the school; how much time can be allocated to a programme like this; what available resources at school could be used; what strategies work best to engage children; who they think should deliver such a programme; what the focus of the programme should be; how to make sure that the programme is relevant and appropriate to children at the school; and whether parents/caregivers should be involved.

We asked school counsellors about their experiences of treating anxiety and depression amongst learners at the school; what they think about implementing the programme with whole classes; what training and/or support they would need to deliver such a programme; what concerns they might have about the implementation of such a programme at the school; who they think should deliver such a programme; if they are familiar with CBT; what their experiences are with parents/caregivers; and what the involvement of parents/caregivers should be in a programme like this.

Participants were provided with light refreshments as a token of appreciation for their time and participation in the study. Interviews lasted between 30 and 45 min and were conducted by trained psychology graduates (SH and HG). Interviews were held in a private room on school premises, and were audio-recorded. Interviews were conducted in either English (*n* = 3) or Afrikaans (*n* = 63), and Afrikaans interviews were translated into English by SH and HG for analysis purposes. All study materials (consent and assent forms, interview schedules and demographic questionnaires) were translated from English into Afrikaans (a local language). Translation of the documents was done by two native Afrikaans speaking members of our research team (SH and HG). Once translation for each of the documents was complete, these translations were checked by a third member of the research team (BC) who is fluent in the language. Given that we did not use standardised psychometric measures in these interviews, we did not back translate these materials. Transcriptions and translations were checked for accuracy by SH and HG.

### Data Analysis

Transcripts were uploaded to ATLAS.ti v8, qualitative software for thematic analysis. We analysed the data according to the six steps of thematic analysis as outlined by Braun and Clarke (2006). Thematic analysis was appropriate given the qualitative and exploratory nature of the work and the broader paradigm of Interpretivism which concerns interpreting or understanding the meanings of individuals’ words or actions within a particular context (Hammersley, 2013). The data were coded inductively by BC, SH and HG, who met frequently to check the consistency and accuracy of the codes developed. For each data set, the data were coded by identifying a quote that was deemed relevant to the aim of the study and this quote was given a code name. Iterations of the synthesis of the results were reviewed by all the authors. The data for each of the groups were analysed separately to explore the unique themes that emerged for each group. Thereafter, instances of similarities and differences were explored across the four data sets and the themes and sub-themes presented across all groups. We ensured rigour and trustworthiness by consulting frequently as a team during the various phases of analysis for consensus on coding and theme development.

### Ethics

This study received ethics approval by Stellenbosch University's Research Ethics Committee: Social, Behavioural and Education Research (project number: 9183) and the Western Cape Education Department (Reference: 20190213–1562). This study received reciprocity from the Psychology Research Ethics Committee at the University of Bath.

### Findings

#### Description of Participants

As given in Table 1, we interviewed 22 CYP who were between 10 and 15 years of age, with a mean age of 11.64 years (SD = 1.00). We also interviewed 21 parent participants who varied greatly in age from 32 to 85 years old (mean = 47.86, SD = 11.59). Thirty-eight per cent of parents (*n* = 8) indicated high school completion as their highest level of education, and 61.9% of the parents (*n* = 13) were a biological parent of the child of interest.

**Table 1:** Demographic information of participants (*N* = 66)

Demographic characteristic	Stakeholder			
	CYP (*n* = 22)	Parents (*n* = 21)	Educators (*n* = 17)	School counsellors (*n* = 6)
*Age in years*				
Mean (SD)	11.64 (1.00)	47.86 (11.59)	38.65 (12.31)	33.17 (8.86)
*Gender*				
Female	17 (77.3%)	18 (85.7%)	7 (41.2%)	6 (100%)
Male	5 (22.7%)	3 (14.3%)	10 (58.8%)	–
*First language*				
Afrikaans	17 (77.3%)	20 (95.2%)	16 (94.1%)	6 (100%)
English	4 (18.2%)	–	1 (5.9%)	–
Afrikaans & English	1 (4.5%)	1 (4.8%)	–	–
*Current grade at school*				
Grade 5	9 (40.9%)	–	–	–
Grade 6	10 (45.5%)	–	–	–
Grade 7	3 (13.6%)	–	–	–
*Relationship to child of interest*				
Biological parent	–	13 (61.9%)	–	–
Guardian/caregiver	–	8 (38.1%)	–	–
*Teaching experience in years*				
Mean (SD)	–	–	13.81 (12.53)	–
*Age range of clients*				
5–15 years old	–	–	–	5 (83.3%)
4–18 years old	–	–	–	1 (16.7%)
*Counselling experience at school*				
1–12 months	–	–	–	3 (50%)
13–24 months	–	–	–	2 (33.3%)
More than 24 months	–	–	–	1 (16.7%)
*Number of hours spent counselling per day*				
2–5 h				4 (66.7%)
More than 5 h				2 (33.3%)

We interviewed 17 teachers who on average, reported 13.81 years of teaching experience. When asked to rate how confident they felt about dealing with mental health issues amongst CYP in their school on a scale from 1 (not confident at all) to 10 (extremely confident), the average rating was 7.44 (SD = 1.49, range 5–10) suggesting teachers felt mostly confident to deal with these issues.

We interviewed six school counsellors. All school counsellors (staff of the NGO in both schools) had completed tertiary education and training in either psychology, educational psychology or social work.

### Results from the Thematic Analysis

As shown in Table 2, we identified three themes and nine sub-themes. Each theme will be described below with illustrative anonymised quotes.

**Table 2:** Themes and sub-themes identified following thematic analysis

Theme	Sub-theme
The value of a mental health and well-being programme	Short term / immediate benefits
	Longer term / wider benefits
Content and delivery	What content should the programme cover?
	How should the programme be delivered?
	Who should deliver the programme?
	Should parents be involved?
Practicalities and logistics	Group versus individual delivery of the programme
	Implementation issues
	Scheduling

### The Value of a Mental Health and Well-Being Programme

#### Short Term / Immediate Benefits

Participants were unanimous about the importance of such a programme in their schools. School counsellors and teachers explained that a programme like this would help them meet the demand for mental health and behavioural support in the schools and assist families who do not have the means to obtain professional help for their children. A teacher stated:

*“…but currently they [school counsellors] are full to the ears uhm so a programme like this where you just focus on it because I just feel those children- I think some of our parents don’t have the means to get professional help for them…”*

However, one teacher had concerns that others would not buy-in to the programme as some teachers believed it difficult to change children’s behaviours. This teacher stated:

*“…they’ll try for a day or two like I said but their their attitude is already “it’s [the programme] not going to work the children are broken they come broken from home so what should I now fix” do you understand they already have this attitude.”*

Participants stated that a programme like this would be beneficial to CYP whose parents work full time and cannot attend to their psychological needs and to CYP whose parents are emotionally absent as a result of alcohol or substance abuse. A teacher stated:

*“... it would be very good for them because they don’t have support at home no one at home can help them they have parents who are on drugs who are alcoholics people who don’t know how to handle their emotions themselves so they can’t help children they can’t give help to their children to handle their emotions they need someone or a programme like that to help them I think it’s very necessary ...”*

CYP reported that participating in such a programme, if taken seriously, might help put an end to bullying and school violence and can help those who have lost a parent or live in difficult circumstances at home. A young person stated:

*“Participant: because they swear .. they fight .. and .. they hit children .. they bully the children .. take the children’s bread .. and stab the children .. with scissors ### knives .. and other things.*

*Interviewer: and do you think such a programme would help that those things happen less often?*

*Participant: yes.”*

#### Longer Term / Wider Benefits

In addition to the more immediate benefits of such a programme, participants were optimistic about longer-term benefits. Participants reported that such a programme could potentially teach CYP a broad range of life skills, including how to cope with feelings, thoughts and behaviours, building self-esteem, problem-solving and goal setting. They thought that this would better equip CYP to handle problems in their future. Indeed, participants explained that if CYP are taught how to handle their feelings, and if their self-image is improved, it could have a positive effect on their schoolwork, and benefit their future. A parent reported:

*“… children will … they will also achieve better in their schoolwork … because look a child who is hurt if that child is sitting with that blow ... in class ... and that teacher is explaining something here on the board and that child’s ... attention is here with the pain ... or with that person who hurt him ... but if they sit in the class with that uhm ... but if they sit with that healthy mind and … they feel happy and so on then they will achieve more ... if they got over that pain and those grudges …”*

Parents reported that a programme such as this is likely to have a ripple effect, in that those CYP who participate in it may ultimately help to reduce problems in the community and can also go tell others about their experiences, driving a larger community benefit of such a programme:

*“… if you can find new ways to help a child for the future it’s fantastic because then there will be less problems in our community …”- Parent participant*

*“… the whole community would benefit from such a programme … we are too negative in our community ... extremely negative ... so the whole community would benefit ... more positive thoughts ... better relationships that people would have in their homes and we’re going to build our community we’re going to uplift it …”- Parent participant*

### Content and Delivery

#### What Content should the Programme Cover?

Participants agreed that a programme focusing on well-being was important and necessary in their context. CYP and parents reported that a programme such as this should focus on and teach them about feelings, emotional development, empowerment, self-confidence and resilience and that this should be done using a wide variety of activities like a puppet show, playing and reading scenarios. A 12 year-old participant stated:

*“… it always helps to talk to people about your feelings and … then you always feel you’re not alone.”*

An 11-year-old participant suggested that:

*“… to expose children more to ... what they can do in their future to build themselves up … to not be shy and scared of things that they don’t need to be scared and to be shy of and ... to be comfortable to talk about yourself and ... activities that can teach you how to build yourself up ... to not bother yourself with things that children say and think of you ...”*

School counsellors recommended that the content of such a programme should be psychoeducational and in-line with the current school-curriculum. They also felt that it should focus on solutions rather than overemphasising mental health problems. School counsellors explained that they thought that focusing on mental health issues might lead CYP to over identify with the issues which could have negative consequences. For example, one school counsellor stated:

*“… with depression and anxiety … you have to be very careful what you say because … you want to address it but you don’t want to over emphasise it … you want to educate the group about mental health ... I think you need to be careful to not over emphasise on emotions but rather on ... what is my future dreams ... what are my strengths ... how can I develop myself ... if there’s an over emphasis on emotions then you’re moving away from problem solving a little bit ...”*

Teachers suggested that such a programme should focus on helping CYP develop hope for the future, understand their own emotions and deal with them in a healthy way. Teachers also suggested that CYP should be taught how to empathise with others:

*“… teach them how do you see someone isn’t well and how should you then react when someone doesn’t feel well … what is the right thing to do are you going to laugh at the person or are you going to say “hi how are you ... what is wrong today can I help you?” … and that popularity doesn’t matter ... you’re a good person or you are mean ... like what is good person you don’t have to be popular ...”*

#### How Should the Programme be Delivered?

Teachers and school counsellors agreed that finding ways to keep children engaged was going to be key to the success of the programme. Based on their own experiences they suggested doing activities that CYP find enjoyable and relatable. They suggested techniques like group work, question and answer sessions, interactive activities, and walking to keep CYP captivated and engaged. They also suggested using visuals and incentives to create a lively presentation style. One school counsellor stated:

*“… things should be visual … if something’s visual and if it’s practical and repetition and another thing that I’ve experienced is that it needs to be suited to their context ... because many times groups of people come to present I’ve seen ... even a simple example that you use can ... lose them completely ... because it’s not in their frame of reference ...”*

One teacher said:

*“… give them an incentive now and then … you get … something like a reading book a diary … pencil bag with crayons … or a ruler they can use in the class ...”*

Participants also mentioned that some care should be taken regarding the possibility of unforeseen/adverse events during the delivery of the programme. Suggestions included establishing clear rules in the classroom and having more than one facilitator to ensure that the programme can continue unhindered.

#### Who Should Deliver the Programme?

Participants reported that the programme be delivered by someone outside of the school. Further, participants agreed that this individual should have sufficient expertise and be someone with whom children would feel comfortable with and can trust. A school counsellor reported:

*“I don’t think it should be internal because .. it helps them if it’s someone from outside and because it I mean it’s delicate it’s not something that children [are] necessarily going to share with a teacher.”*

Participants reported that the presenter or facilitator of the programme would need to make every effort to maintain discipline in the class. A young person stated:

*“because many children they won’t take notice.. they will just talk to friends.. then they make jokes about it.. and things.”*

Teachers reported that their presence in classes during the delivery of the programme is likely to help the facilitator maintain discipline. However, some teachers were concerned that their presence in the class may deter some CYP from participating freely and openly. A teacher stated:

*“…like with discipline for example but in cases where it’s more uhm personal I don’t believe the educator should be involved.”*

#### Should Parents be Involved?

Participants spoke about the extent to which parents should be involved in the programme. While some participants felt parents should certainly be informed of such a programme being delivered to their children and that this would help reinforce the content, they also recognised that involving them directly may be difficult. An teacher stated:

*“…to be honest .. especially with the children who really need it .. but it’s difficult the get their parent .. involved .. but to uhm complete the triangle you need the parents .. understand because the support system works in threes the learner the support and then the parent because if the parent isn’t going to be involved then the child is going to .. go through the same feelings at home but no one is going to be there to support him if such things happen so I would say the parent .. should be involved but the .. way to get the parent involved is very difficult .. yes”*

Participants stated that some parents might lack the skills and knowledge to engage with their children in this manner and that some form of parent training could therefore be beneficial. Others were concerned that parents would not have the patience to become involved. As one parent reported:

*“…because it’s already a challenge with my own [child] because sometimes I feel I want to go uhm .. auction them somewhere and then they can come back again when they are grown up (laughs) .. and earned money .. otherwise .. you have to have patience and sometimes my patience just isn’t that much (laughs).”*

Parents reported that becoming involved in such a programme would provide them with an opportunity to further build a relationship with their child, and to understand their children better. Parents reported that they too would benefit from such a programme as many of them need this sort of support as well. A parent remarked:

*“…it needs to be a joint thing that the children also feel that .. they can open up to their parents now .. “mm .. okay .. I told auntie .. uhm .. in my sessions .. mommy is this and mommy does this .. and daddy does that wrong .. that’s wrong or the sibling does this wrong .. but .. how am I going to tell them .. without hurting them and to say how I felt” .. so as soon as they open up in certain sessions .. that people should get involved .. is how I feel .. they need to get the same uh .. guidance .. the same .. assistance .. so that they can grow and they .. they grow in the process .. and that they could also help the children .. so yes .. I .. I really think everybody needs to get involved.”*

### Practicalities and Logistics

#### Group Versus Individual Delivery of the Programme

Participants reported that whole-class delivery was possible but felt that it could be hindered by a lack of interest from CYP, discipline issues, and a lack of sensitivity among CYP. A young person stated:

*“let’s say because- there’s some people that would talk about you to their mothers to their friends or to other people in the school and then it spreads around”*

School counsellors reported that delivering the intervention to the whole class would promote accountability amongst the learners and to the programme content, while parents were convinced that large groups would provide their children with peer support. A parent reported:

*“… at their age peer pressure … can become a big problem if it’s if it goes into a negative direction you know and all of them together being this kind of like safety becoming a safety net for each other during the day …”*

School counsellors suggested that keeping the mixed gender dynamic within the classroom was important and would facilitate a familiar context, while others expressed concern. The latter expressed that as teenagers, the two genders are “over-aware” of one-another, which may interfere with their willingness to be open and honest. A school counsellor stated:

*“I think the girls boys they experience things that they wouldn’t necessarily want to say in front of the other or the boys are going to be too cool to say in front of the girls (..) I would think it would be better to separate the girls and boys .. because especially maybe not grade 5 as bad but I think grade 6 and 7 I have- they’re awful the boys irritate the girls and the boys have to be these macho guys in front of the girls”.*

#### Implementation Issues

Teachers reported that due to the large number of learners per class and limited contact time, they struggled to give individual attention to CYP who needed emotional support. Due to these issues, participants suggested that the programme should be implemented with smaller groups consisting of no more than 10 to 12 CYP. A parent stated:

*“if you can maybe work in smaller groups because look sometimes children can also be cruel sometimes they see for example you’re going through this thing and you’re getting counselling for it and so on- they- you get that child who’s going to tell ‘did you hear about [child’s name omitted] thing’ (…) and like that so I would rather say uhm work in smaller groups then you can- then you can also give more attention because if you’re in a big group for example here in a classroom with 42 children you can’t give individual attention to every child.”*

Other suggestions to facilitate the implementation of the programme included, having more than one facilitator in the classroom when implementing the programme, building rapport with the learners, making use of a reward/punishment system to teach CYP the consequences of their actions, and keeping to rules and schedules. A teacher stated:

*“.. because you have those 2 or 3 children who has the ability to disrupt a whole class where the rest of the class actually show good behaviour so uhm it can it can work if uh and I think what is very important is that the structure and the rules are made very clear to them beforehand ..”*

#### Scheduling

Participants were concerned that scheduling such a programme during school hours would interfere with already limited teaching time. Participants also stated that scheduling the programme after school hours might bring about issues regarding motivating CYP to attend the sessions, coordinating with other extra-curricular activities, and school transport. A teacher stated:

*“within contact time it can be done but it’s going to be difficult and I think at the end of the day it’s going to- the educators are going to suffer because of it because uhm our curriculum expects that we need to do so much without time already without any extra-curricular is already too little for all those things that we have to do so if we implement a programme inside of contact time and we still do the uh we have to look at the .. the performance that also needs to take place that is also taking up uh time it’s going to be very difficult to run such a programme- or to run such a programme efficiently within school hours”*

Despite these concerns, teachers were mostly accommodating concerning the scheduling of such a programme and were willing to negotiate time for its implementation, given its importance. Most teachers suggested that one 25–30 min class period per week (at maximum two) would be feasible. Participants were not willing to sacrifice time spent on subjects like Mathematics and Languages but suggested that the programme could be accommodated within the time allocated for Life Orientation classes.

## Discussion

Our participants reported an urgent need for a universal mental health programme for CYP within a South African primary school setting, which they thought could help CYP to develop coping skills and resilience. The need for such a programme was further emphasised in the context of poor parental support at home and believed to have possible benefits for educational performance too. Participants also thought it could have potential protective effects into adulthood. Participants thought that the programme should focus on emotional development and empowerment, with an emphasis on feelings, building self-esteem, problem-solving, empathy for others, future goals and instilling hope. Participants highlighted the need to engage CYP through an interesting and interactive approach which is relevant to them, and to manage any adverse events should they arise. Generally, participants were in favour of the programme being delivered by staff who were external to the school and who had expertise in working with young people. While whole-class delivery was seen as a good approach, the issues of discipline and peer pressure, as well as gender issues were raised by some. A potential solution suggested was breaking into smaller groups to do activities. While participants thought it would be hard to accommodate the time for a programme within the other pressures of the timetable, time allocated to Life Orientation classes was seen as a potential opportunity to do so.

It is encouraging that all stakeholders saw the need for a universal, school-based mental health programme, particularly in the context of heightened vulnerability to developing mental health problems in the South African context in which access to specialist services is limited and costly (Patel & Kleinman, 2003). While some of the potential anticipated benefits were direct and measurable, such as emotional literacy, others will be harder to track over time, such as long-term mental health outcomes into adulthood, and spill over effects at community level, and consideration should be given to how best to evidence these more intangible benefits, particularly to ensure that cost-effectiveness calculations for future trials capture the breadth and potential longevity of the benefits of such a programme.

CYP particularly emphasised the need for a programme to focus on feelings. The cognitive behavioural model assumes that changes in patterns of thinking and doing can lead to changes in feelings due to the interconnected nature of thoughts, feelings and behaviours (Stallard, 2002). The cognitive behavioural model has predominantly been developed and tested in populations in high-income countries. Therefore, it may be that the model does not necessarily fit in this cultural context (Rosenstein & Seedat, 2011), although CBT principles were used in several previous prevention programmes in LMICs (Bradshaw et al., 2021) and the goal-focused, skill-based approach does appear to fit well with what is required. At the outset of CBT, it is important to socialise CYP to the model, helping them to become familiar with the underpinning principles (Mahoney-Davies et al., 2017). It may be that their emphasis on feelings reflects their desire to change feelings, and a programme needs to help them to understand that this can be done via changing thinking and doing.

It was interesting that neither CYP nor teachers thought that teachers should deliver the programme. Teachers’ concerns were largely based around issues with scheduling of the programme delivery and perhaps confidence to effectively deliver the sessions, despite self-reporting that they feel confident in dealing with mental health issues. This confidence issue with delivering the sessions may be more around the practicalities of having to deliver a programme when already feeling over-stretched by their teaching load and other demands. Both teachers and CYP also believed that teachers being present in the classroom may deter CYP from engagement and openness. In other settings, like the United Kingdom, teachers have also expressed reluctance about delivering programmes, specifically CBT-based prevention programmes, although for different reasons. For example, in one study, teachers expressed that there were too many strategies to cover within the allocated time, and that the content was not sufficiently different from what was routinely offered within the curriculum (Skryabina et al., 2016).

Participants, including parents themselves, also commented on parental involvement in such programmes. While parents had concerns about time availability, they maintained a general optimism towards being involved as they valued not only the benefits it would hold for their children but also for themselves. In South Africa, with an unemployment rate of nearly 30% (Plecher, 2020) and where a large proportion of the population rely on a daily wage, and shift-based work, parental involvement may be challenging and difficult to prioritise. Indeed, these challenges seem universal with regards to parental involvement in mental health interventions (Gee et al., 2020).

### Strengths and Limitations

We interviewed a range of CYP, parents and professionals, including teachers and school counsellors in two schools. Given the importance of stakeholder buy-in for the success of such a programme, interviewing all of these participants is a major strength of this work and likely to afford us with the opportunity to assess long-term outcomes which is missing from prevention work. While the aim of qualitative research is not to strive for generalisability of findings, it is important to consider how our findings might be generalisable to other similar contexts, i.e. transferability (Smith, 2018). As such, our findings may be transferable (that is, of interest to and important to) to researchers working in settings like ours who are exploring universal prevention interventions in school settings. Further, our collaboration with the NGO which provides psychosocial support to learners in these schools is likely to hold long-term benefits and there is evidence to suggest that schools partnering with mental health organisations are more likely to experience successful implementation of outside programmes (Langley et al., 2010). However, it is likely that our sample of CYP and parent participants is biased by those who may be familiar with the NGO and their services in schools. As such, CYP and their parents who do not access these services may not have participated. Further, parent interviews took place during working hours and this may have excluded parents who work full time.

### Implications

Our findings suggest that the content and delivery of such a universal intervention requires an understanding of what would keep CYP engaged in a programme that may be emotionally burdensome and require of them to potentially disclose difficult and uncomfortable thoughts, feelings and behaviours. In an individual therapeutic setting this may be possible, but amongst CYP in our sample, disclosing difficult emotions to their peers and potentially their teachers within the context of a whole-class approach would discourage them from participation. As such, within this context, developing a prevention intervention that is positively oriented to focusing on resilience and life skills such as self-esteem and problem-solving may be better suited. Further, ensuring engagement would require sessions that are interactive and fun and perhaps even rewarding in nature.

A concern raised by participants was about behaviour management in the classroom context, with both counsellors and teachers suggesting that teachers maintain a presence in the classroom to ensure discipline. Identifying discipline as a potential barrier to programme delivery is important when one considers that in South Africa a public school classroom typically hosts 40–45 learners at a time, with one teacher to facilitate the learning needs of them all. As suggested by participants in our study, to ensure delivery and to adhere to potential time restrictions for delivery, several facilitators within one classroom would be needed to facilitate smaller groups within the classroom. Further to ensure fit within the school schedule and to mitigate timing concerns, the programme would need to be flexible, with sessions preferably brief and easily adaptable.

## Conclusions

In the absence of sufficient mental health resources and services to address the mental health needs of CYP in South Africa, universal mental health prevention programmes may help to mitigate the onset of disorders by building coping skills and resilience. Schools hold potential as suitable locations for these interventions and our findings suggest that implementing such interventions may well be possible if practical and logistical difficulties are addressed. Further, to ensure sufficient buy-in and intervention effectiveness at long-term follow-up, stakeholder roles in these interventions need to be carefully assessed.

## Data Availability

Data are available on a secured repository. For ethical reasons these data are not available publicly.
